# Evaluation of kikuyu grass wetland for wastewater treatment efficiency from malt factory, Ethiopia

**DOI:** 10.1186/s12889-024-19496-5

**Published:** 2024-07-29

**Authors:** Dessie Tibebe, G/ Hiwot Ambelu, Yezbie Kassa

**Affiliations:** 1 Department of Chemistry, University of Gondar, P. Box 196, Gondar, Ethiopia; 2 Department of Biology, University of Gondar, P. Box 196, Gondar, Ethiopia

**Keywords:** Gondar, Malt factory, Removal efficiency, Treatment, Wastewater, Wetland

## Abstract

**Background:**

The majority of existing industries in Ethiopia discharge untreated effluents into nearby water bodies, streams and open land. The wastewater generated by Gondar malt factory (GMF) was disposed freely and join a natural wetland implemented for the treatment of the wastewater. The objective of the study was to analyze and characterize wastewater from GMF and to evaluate the effectiveness of the wetland for the treatment purpose.

**Methods:**

Different Physicochemical quality indicators (color, turbidity pH, temperature, Total Dissolved solids, Total Suspended solids, total solid, conductivity, alkalinity, hardness, nitrate, phosphate, sulfate, free chlorides, heavy metals-(Cd, Cr, Fe, Mn, and Pb)and Biological Oxygen Demand were measured according to the standard procedures. Data was analyzed using Statistical Package for Social Sciences (SPSS-25). Analysis of Variance (ANOVA) was used to find whether significant differences existed in the different sampling stations for the parameters studied. P value less than 0.05 was considered to show significant difference.

**Results:**

The results of this study revealed that most of the quality indicators were improved in value after the water passed through the wetland except for alkalinity M, sulfite, Mn, temperature and pH. From ANOVA result, it was noted that there was a significant mean difference between the stations except for chromium, manganese and lead. The result showed that the wetland plays a great role in the removal of pollutants where the best performance was obtained at removal efficiency of 96.188% PO_4_^HR^_,_75.63% Nitrate,>99% Cl_2,_ ammonia and nitrite 99.99%, 92.77% sulfate,84.36% Total hardness,87.43% color, and for others it is ranged between 30 and 60%.

**Conclusion:**

the study concluded that GMF wetland was almost effective and had potential in treatment of the wastewater from the discharging facilities (especially for nutrients, alkalinity P, hardness, color and chloride). It is recommended that wetlands should be conserved and used as wastewater treatment facility.

## Introduction

Environmentally comfortable waste management system is among the tasks of great concern [1]. To provide better, faster, cost-effective and cleaner method of treating effluent, a number of parameters and factors need to be studied, analyzed and treated. This is possible through characterization of the type and nature of the effluent generated. Now a day, the brewing and malting industries have shown increasing awareness for environmental protection and the need for sustainable production process. Currently, knowledge about environmental problems is becoming management information, which may help to improve the efficiency of in-plant malting processes. The majority (90%) of existing industries in Ethiopia discharge untreated effluents into nearby water bodies, streams and open land [2]. It is well known that in most developing countries, industries dispose of their effluents without adequate characterization, quantification and pretreatment due to economic and technological constraints [3]. Being a factory in developing countries, GMF is not exceptional.

In malting process, the barley grain embryo will only grow when immersed in water, which is done through the ***steeping*** process. During steeping between 0.5% and 1.5% of the grain’s dry weight dissolves in the steep water. Steeping a 100 tone batch of dried barley can add around 1 tone of solids into the total volume of effluent from the batch (over the 2 or 3 wets during steeping). Biological oxygen demand (BOD) increases with the length of contact time between grain and steep water. Larger volumes of water result in more dilute effluent. On average for every tone of malt produced 4.5–5.0 m^3^ water is required for the steeping process and the wastewater generated is approximately two thirds of this [4]. A Biological (wetland) effluent treatment processes have been applied to a number of different wastewaters including: the food processing, wine making, beer brewing and animal slaughtering industries. These processes have long been used successfully to treat effluents of food industry [5].

The ability of wetlands to improve the quality of water has long been recognized and this had led to proliferation of wetlands as a means to treat diffuse and point source pollutants from a range of land uses. This is mainly done in the temperate climate, with paucity of information on the effectiveness of wetlands particularly natural wetlands in tropical regions, where the practice of discharging waste water into natural wetlands has been used as waste depository for hundreds of years [6]. The effectiveness of the wetland in treatment of the wastewater is limited yet; most companies and human activities release their waste into the wetlands.

In the past times the wastewater generated by Gondar malt factory is disposed freely and join the nearby water body (Shinta and Dimaza-as the end Megech and goes to Lake Tana being its watershed without any treatment. Communities downstream of Gondar Malt Factory use the water from the river in which the effluent is part, for a variety of purposes such as drinking, livestock watering, irrigation, and other purposes. Besides, the bad smell released from the wastewater canal has created discomfort to the extent that the nearby people and administrators are claiming and expressing their worry. Hence, the factory prepares wetland to treat the waste before a year. Result of this research is believed as one key input to show (determine) the wastewater concentration and contribute to the evaluation of the natural wetland and also background information for the planned expansion work.

Organized information on the wetland as wastewater treatment plants is missing. GMF wetland in Gondar is a small size wetland that might be faced with extinction due to land pressure and overloading with waste. It is currently assumed that the GMF wetland retains the nutrients, chemicals and pathogens carried with the wastewater, but there is no quantification of this function. Such information is necessary for the efficient planning of long-term sustainable use of the wetland. Hence, this study is designed to characterize the wastewater and evaluate the effectiveness of wetland in the malt factory for treatment of the wastewater flowing through it.

## Materials and methods

### Description of the study area

**Fig. 1:**
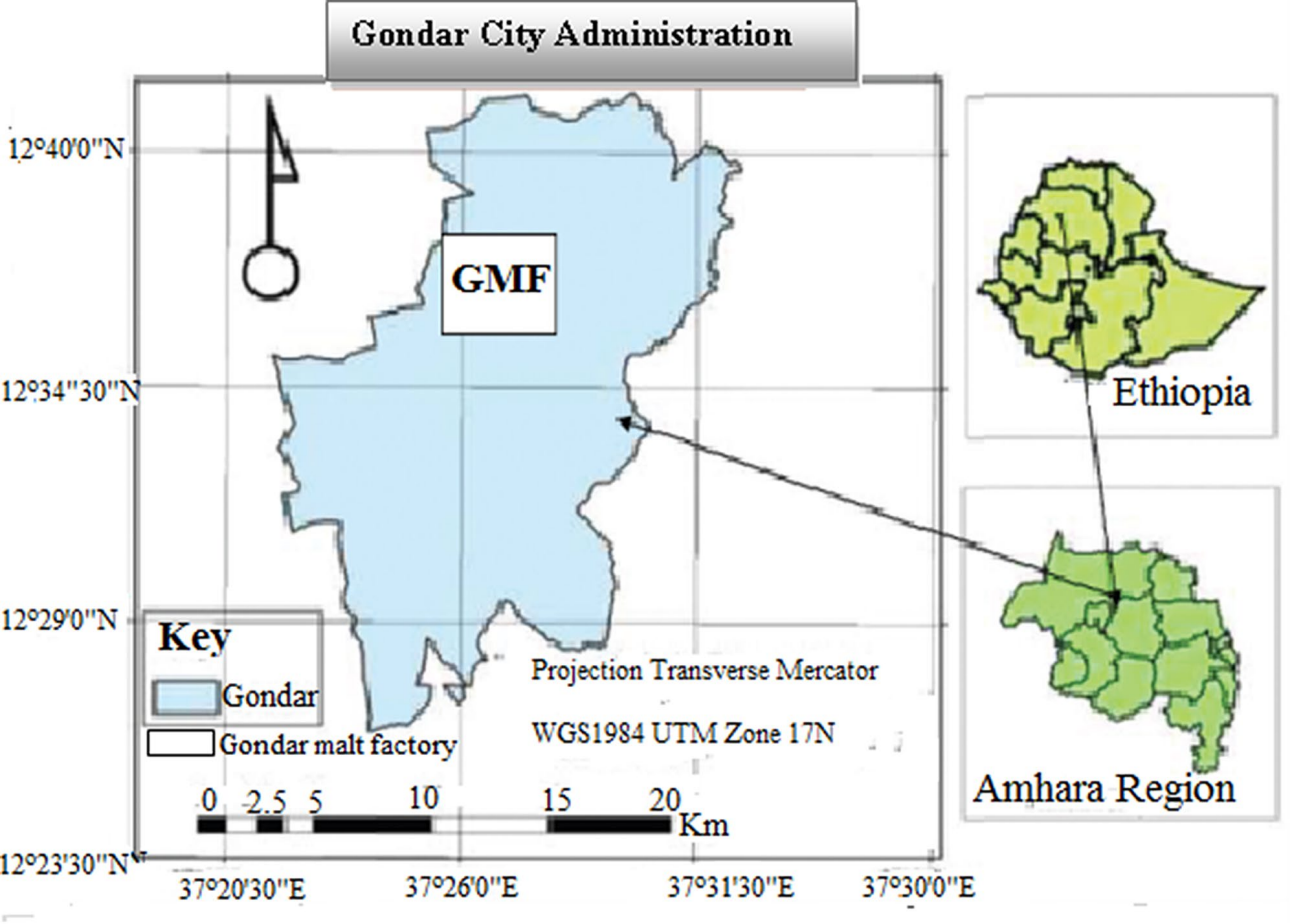
Map showing the location of Gondar malt factory (GMF) in Gondar City, Amhara region, Ethiopia [7]

This study was conducted in Gondar town, which is the capital city of central Gondar zone and it is located 747 km North West of Addis Ababa and 180 km North East of Bihar Dar. It is located at 12,030’ North and 37,020’ East. The town limits of Gondar enclose an area of 48.27 km^2^ and standard altitude is 1966 m above sea level [2]. Gondar malt Factory was established in 2006 June 26 E.C. and currently it is the second supplier of malt in the market with production capacity of 172,000 quintal malt per annum. The factory is located in the east-western part of Gondar city (Fig. 1).

**Fig. 2:**
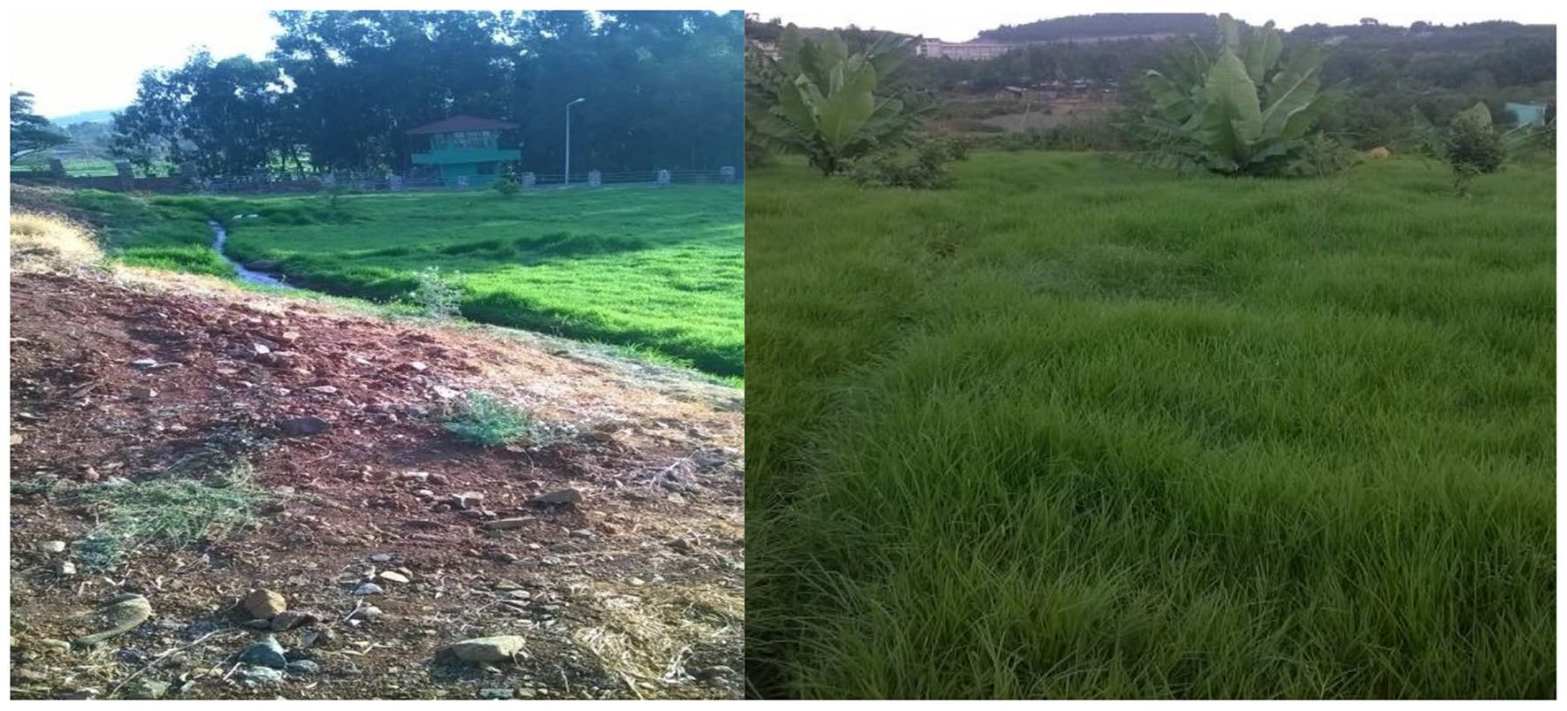
Downstream of GMF

The wastewater generated by Gondar malt factory is disposed freely to the GMF wetland implemented for the treatment of the wastewater. GMF is a small size natural wetland which directly connected with Shinta River (Fig. 2).

River Shinta which contribute to Megech river part of the water shed of Lake Tana is located near from the factory and it drains the wastewater from the wetland along with other sources from north to south and it serve as natural sewerage lines for domestic and industrial wastes.

It is currently assumed that the GMF wetland retains the nutrients, chemicals and pathogens carried with the wastewater, but there is no quantification of this function. Such information is necessary for the efficient planning of long-term sustainable use of the wetland.

### Sampling design

Wastewater samples were collected from 4 sampling stations, one sampling station (Station 1) was located at the junction, where the fresh water from the storage enters to the steep tank for steeping barley. Sampling stations 2 and 3 (for 1st and 2nd wet) along the course of the effluent discharging canal which was located at the downstream location; at 30 m from the steep tank. The other sampling station (Station 4) was located along the wetland, 10 m from the point of discharge into the river.

### Sample procedures and analysis

Wastewater samples were collected in triplicate at the 4 sampling stations along the course of the water and the waste discharging from June 2018 to September 2018. Water samples were collected in a plastic bottle which was thoroughly cleaned by distilled water. The samples were taken to laboratory immediately for analysis. The physicochemical parameters studied were color, turbidity pH, temperature, Total Dissolved solids (TDS), Total Suspended solids (TSS) total solid (TS), conductivity, alkalinity, hardness, nitrate, phosphate, sulfate, free chlorides, heavy metals-(Cd, Cr, Fe, Mn, and Pb) and Biological Oxygen Demand (BOD). pH of samples was measured using pH meter (HI-111, Egypt) already standardized by using buffer solutions of known value (4 ,7 and 10) before analyses [8]. The Palintest Alkaphot M and Alkaphot P tests were used as it provides a simple means of checking Alkalinity M and Alkalinity P levels over the range 0 to 500 mg/L CaCO_3_. Temperature, conductivity and Total Dissolved Solids (TDS) of the water were measured using HACH Track instrument (conductivity meter HQ14d, Germany) [8]. Biological Oxygen Demand (BOD) was measured according to a 5 day test method using BOD instrument (DR/2010 HACH, Loveland, USA) according to the HACH instruction. What man No.1 filter paper was used to determine samples TSS and TS [8], heavy metals were determined using AAS and others were using photometer 7100 according to the standard methods for determination in APHA, 2005 [9].

### Data analysis

Data was analyzed using Statistical Package for Social Sciences (SPSS-25). Analysis of Variance (ANOVA) was used to find whether significant differences existed in the different sampling stations for the parameters studied. P value less than 0.05 was considered to show significant difference.

## Results and discussion

This study has shown the current conditions of GMF effluent on the nutrient, metal and physicochemical parameters. Results and % removal efficiency of the different parameters of Gondar malt factory fresh water, wastewater at the 4 sampling site is shown in the table below (Table 1).

**Table 1:** The mean values and % removal efficiency of the physical parameters of water quality

Parameters	Sample site				% Removal efficiency
	1(freshwater) water) mean	2(1st wet) mean	3(2nd wet) mean	3(effluent) mean	
Alkalinity m	233.33	418.30	284.12	570 0.00	-36.26
Alkalinity p	46.67	70.67	45.00	30.00	57.54
Ammonia	ND	0.56	ND	ND	> 99.99
BOD	45.00	732.00	330.00	465.00	36.48
Color	BDL	6205.56	2903.33	780.00	87.43
Conductivity	110.10	1108.33	416.33	749.00	32.42
Cl2 free	0.58	6.61	1.79	0.06	99.09
Nitrate	0.09	3.57	1.55	0.87	75.63
Nitrite	0.00	0.27	0.03	0	> 99.99
pH	8.59	7.17	6.55	7.97	-11.22
Phosphate ^HR^	18.87	404.06	47.49	15.40	96.19
Phosphate ^LR^	0.46	7.22	0.87	2.96	59.00
Sulfate	10.33	49.67	4.33	6.00	92.77
Sulfite	13.00	8.00	18.33	18.00	-125.00
TDS	55.10	557.67	213.73	381.00	34.05
Temperature	18.00	22.47	21.97	23.00	-2.37
Total Hardness	81.67	287.78	145.00	45.00	84.36
Total Solid	55.10	774.33	335.07	497.00	35.81
TSS	BDL	196.67	121.33	116.00	41.02
Turbidity	BDL	167.89	120	80.00	52.35
Cd	BDL	0.11	0.0267		
Cr	BDL	0.21	0.167		
Fe	0.03	1.35	0.94	0.95	29.80
Mn	0.00	0.01	0.001	0.03	-125.56
Pb	0.00	0.00	0.00176		

*N.B*: *BDL* Below detection limit

Temperature: Water temperature was varied from 18 ^o^C to 23 ^o^C and comparatively the lowest temperature was recorded in Station 1 (18^o^C) and the highest was recorded for Station 3 (effluent). The relative high temperature recorded for station 3 could be attributed to discharges of hot liquor and steam condensates from the malting operation [10]. The temperature range of the effluent (18–230 C) was within the EEPA (2003) [11, 12] and World Bank (2007) [13] permissible limits of < 30 ^o^C [13]. Using the t-test, it was noted that there was no significant difference (*P* > 0.05) mean temperature between the inlet (1st ) and after passing through the wetland (effluent). Water temperature was influenced by time of the day, season and presence of vegetation and amount of dissolved solids [14]. The higher temperature in the wetland could be attributed to high amount of organic matter (BOD) and TDS (Table 1). The decomposition of organic matter produces heat while the TDS absorbs heat from the sun and this could be responsible for the higher temperature in the wetland. The lower temperature at the inlet could be attributed to the cooling effect and decreased organic matter concentration (Table 1).

pH: The pH range for this study was 6.55 to 8.59. Comparatively Station 2 (2nd ) had the lowest values and Station 1 (fresh water) had the highest pH values. Statistical analysis using ANOVA (T-test) showed that pH value was not significantly different (*p* > 0.05) between the inlet and outlet stations sampled. The pH range recorded in the study was in line with EEPA and World Bank (2007) [13] permissible limit of 6–9. The average value of pH of the GMF effluent was 7.97, this result is in line with the WHO permissible limit of 6.5–8.5 [11, 12, and 13]. In similar work on the brewery, a wide range of pH values in wastewater discharged from different sections of the industry was reported [15] This difference in pH of wastewater might be due to the nature of brewery batch processing and the usage of different chemicals (NaOH, H3PO4,HNO3) used in cleaning in place (CIP) units.

Total suspended solids (TSS): This study showed that the values of total suspended solids (TSS) fluctuated between BDL and 196.67 mg/L where station 2 (1st wet) had the highest mean value and the lowest mean value was recorded for Station 1 (freshwater). Statistical analysis using ANOVA (t-test (2-tailed analysis) indicated that there is significant mean difference (*p* < 0.05) in the value of TSS between the wastewater inter to the wetland and water leaving the wetland. The highest TSS value at Station 2 could be attributed to effluent contains organic materials like spent grains, waste, grit and draining of effluent discharges with high TSS levels into the wetland [16]; [17] also reported similar findings. The decrease in TSS concentration noted at the outlet can be attributed to the luxuriant vegetation which traps some of the solids as well as reduced water velocity as the water flows through the wetland hence causing most of the suspended solids to settle from within the water column on the grass. The mean TSS value of the effluent i.e. 46 mg/L is within permissible limits 50 mg/L set by WHO [11–13]. Moreover, the variation in concentration of Suspended solids was attributed to runoff or inflow from associated water bodies [18]. Low velocity coupled with the presence of the luxuriant vegetation and gravel substrate also contributed to lower TSS [19]. Removal of other pollutants like BOD, COD, and heavy metals from the water also leads to decrease in TSS concentration. Erosion, urban runoff and agricultural land, industrial wastes, bank erosion, bottom feeders, algal growth or wastewater discharges increase TSS in water [20].

Total dissolved solids (TDS) and total solids (TS): The mean values of Total dissolved solids (TDS) and total solids (TS) were found in the range of 55.1 to 557.67 mg|L and 55.1 to 774.334 mg|L respectively (Table 1).The mean values for both solids TDS and TS were also significantly different (*p* < 0.05) among the stations sampled where station 1 (Fresh water) recorded the lowest mean value and station 2 (1st ) recorded the highest mean value (Table 1). Statistical analysis using t-test (2-tailed analysis) found that there is a significant difference between both TDS and TS concentration in the wastewater inter to wetland and after passing through the wetland (*p* < 0.05). Variations in concentration of Total Dissolved Solids might be attributed to presence of organic matter, runoff, from urban areas road salt use in street fertilizers and pesticides used in farms. Inorganic materials and air that contains calcium bicarbonate, nitrogen, iron phosphorous, sulfur and other minerals [20], Fertilizer, decaying of plants and animals, solids and ions deposition onto wetland. The decrease in TDS noted at the outlet could be attributed to solid deposition due to reduced water speed as the water passes through the wetland. The decrease could also be attributed to uptake of some of the dissolved solids by wetland plants.

Higher concentration of TS in the wetland could be attributed to Industrial discharges from GMF. The decrease in the concentration of TS at wetland outlet might be as a result of reduced velocity of the waste water as it flows through the wetland due to the luxuriant wetland vegetation hence causing the solids to settle at the bottom or be attached on the roots of the wetland vegetation’s.

Biological Oxygen Demand (BOD): The result of this study showed that Biological Oxygen Demand (BOD) fluctuated between 45 and 732 mg/L where the fresh water at station 1 had the lower value however, Station 2(1st wet) had the highest value (Table 1) and analysis of variance (ANOVA) result showed that BOD values are significantly different (*p* < 0.05) among the sampling stations. The increase in BOD value of the station 2 can be attributed to an organic loading of the effluent discharge and this was in accordance with the fact that high organic load was found in wastewaters of malt from steeping of barley and washing tanks, which consequently affect high bacterial population [16]. The variation in BOD concentration between the inlet and the outlet might also be attributed to surface runoff and natural biotic processes [18]. Wetland vegetation, decomposing microrganisms and temperature also influence the BOD in a water body [21, 22]. The mean BOD level of the effluent (465 mg/L) is higher than the EEPA and World Bank 2007 permissible limit of 60 and 50 mg/L respectively [13]. The note worth reduction in BOD concentration at the outlet can be attributed to biodegradation of the organic matter by microorganisms in the wetland. The trapping of particulate organic matter by wetland vegetation, might have also contributed to decrease in BOD concentration at the outlet as the organic matter settle as sediment off the water column.

Alkalinity: The Palintest Alkaphot M and Alkaphot P tests provide a simple means of checking Alkalinity M and Alkalinity P levels over the range 0 to 500 mg/L CaCO_3_. Results of this study showed that as Alkalinity m fluctuated between 233 and 570 where the wet station 1 (fresh water) had lower values, and station 3(effluent) had relatively high value. And Alkalinity p fluctuated between 30 and 70 where the station2 (1st wet) had the higher value and station 3(effluent) had relatively lower (Table 1). Analysis of variance (ANOVA) result showed that alkalinity were significantly different (*p* < 0.05) among the sampling stations.

This might also be due to the presence of T-cera detergent for cleaning purposes. However, the alkalinity values of RW were lower than the allowable limit of 200 mg/L. In the absence of an alternate wastewater source, an acceptable alkalinity level of up to 600 mg/L for drinking wastewater could be tolerated [8]. In this study, the high overall mean values of alkalinity could be the presence of -carbonate-containing compounds. The pattern was as follows: WD *>* WAW *>* RW.

Ammonia: The value of ammonia was detected only in the station2 (1st wet) with the value of 0.57 mg/l. N, but not from other sampling stations (Table 1). In this study color ranges from BDL to 6205.57 mg/L Pt, where station 1(fresh water) had BDL value and station 2(1st wet) had highest value 6205.57 mg/L Pt. The t-test (2-tailed) analysis found that the color were significantly different (*p* < 0.05) among the 2 stations sampled.

Conductivity: Conductivity ranged from 110.10 to 1108.33 µs/cm where station 1(fresh water) had 110.10 value and station 2(1st wet) had highest value 1108.33. Conductivity in wastewater is attributed to the presence of negative and positive ions [18, 23], the amount of total dissolved solids, total suspended solids and diatomic nitrates in water [24]. Wetland plants also influences conductivity of water as they trap sediments and pollutants [25]. T-Test (2-tailed analysis) showed that the conductivity were significantly different (*p* < 0.05) among the 2 stations sampled. The difference in conductivity in water before and after passing through the wetland could be attributed to the fact that the wastewater before it enters the wetland has high concentration of TDS and TSS .The TDS and TSS settles at the bottom of the wetland in the form of sediment as the water passes through the wetland and this causes a decrease in conductivity. The TSS might be trapped by plants as the water passes through the wetland and this result in decrease in conductivity as the water leaves the wetland. The decrease could also be attributed to conversion of NO_3_-N into diatomic molecular nitrogen (N_2_) as the concentration of NO_3_-N decreases (Table 1). Similar results has been seen in the studies done on treatment performance assessment of natural and constructed wetlands on wastewater from kege coffee processing plant [26].

Chloride: The study has also showed as free Chloride fluctuated between 0.06 and 6.61 mg/L Cl where station 2(1st wet) had the highest value and station 3(effluent) had the lower value. ANOVA t-test (2-tailed analysis) showed that the chlorine were significantly different (*p* < 0.05) among the 2 stations sampled.

Nitrate and Nitrite: Similarly, Nitrate fluctuated between 0.87 and 3.57 mg/L N where station 3(effluent) had lowest value and station 2(1st wet) had relatively the highest value. Using T-Test (2-tailed analysis) it was found that the concentration of nitrate were significantly different (*p* < 0.05) among the 2 stations sampled. Moreover, it was observed that Nitrite fluctuated between BDL and 0.27 mg/L N. The station 3(effluent) had the BDL value and Station 2 (1st ) had the highest value. Using T-Test (2-tailed analysis) it was found that result showed that the nitrite were significantly different (*p* < 0.05) among the 2 stations sampled.

Variations in concentration of nitrate-nitrogen were attributed to runoff from vegetated watershed, effluent discharge and runoff from fertilized crop land [18, Denitrification [27–30], uptake by vascular plants and subsequent burial when the plants die. The high level of nitrate-nitrogen concentration recorded in the waste could be attributed to wastewater from Company which contains high organic matter, while the low nitrate-nitrogen concentration recorded at the wetland outlet could be attributed to denitrification where nitrate was converted to diatomic molecular nitrogen, deposition of nitrate in sediments at the wetland bottom and plant uptake.

Phosphate : The other nutrient studied was phosphate whose values were fluctuated from 15.4 to 404.06 mg/L PO_4_ and 2.96 to 7.22 mg/L.PO_4_ for Phosphate ^HR^ and Phosphate ^LR^ respectively where the station 3(effluent) had lowest value and station 2(1st ) had the highest value in both cases. ANOVA result (2-tailed analysis) showed that both Phosphate ^HR^ and Phosphate ^LR^ were significantly different (*p* < 0.05) among the two stations sampled. From effluent characterization work the source of phosphorus is found to be at the first steeping process where washing and cleaning of grains of different kernel size took place using the chemical Phostoxin as a disinfectant to prevent grain damage by pests in storage silos.

The composite effluent at the outlet has relatively very small component of phosphorus while its concentration was high in the first and second wet steep. The reason for this could be attributed to the nature of phosphate itself to be adsorbed on the surface of colloidal matters and suspended solids and became removed from the liquid stream. It can be also attributed to runoff, effluent discharge and runoff from fertilized cropland and adsorption of phosphates onto mineral sediments [21]. The presence of Ca^2+^, Fe^3+^ or Al^3+^ in Sediments can also determine adsorption capacity [31]. Physical, chemical and biological processes [28]. Precipitations with metal oxides to form new mineral compounds are also the probable sources of it [32].

Sulfate and Sulfite: Sulfate fluctuated between 6.00 and 49.67 mg/L SO_4_ in station 3 (effluent) had lower value and Station 2(1st ) had relatively high value as seen in the Table above (Table 1), the amount of sulphate is high in the wastewater; the source of sulphate could be aluminum sulphate used for inward water treatment of the factory main inlet water treatment plant. Using T-Test (2-tailed analysis) it was found that the sulfate were significantly different (*p* < 0.05) among the two stations sampled. And sulfite fluctuated between 8 and 18 mg/L where station2 (1st wet) had lower value and station 2(2nd wet) and station 3(effluent) had relatively the same high values of mean 18 mg/L Na_2_SO_3_ (Table 1). Statistical Analysis t-test (2-tailed analysis) showed that sulfite were significantly different (*p* < 0.05) among the two stations sampled.

Total hardness and Turbidity: The other parameters studied were Total hardness and Turbidity which were fluctuated between 45.00 and 287.76 mg/L CaCO_3_,and 80.00 and 167.89 FTU respectively where station 3(effluent) had lower values and Station 2 had relatively high values for both cases (Table 1). Similarly significant differences (*p* < 0.05) were observed between the two sampling stations for both.

Metals: The range of Cadmium in this study fluctuated between BDL and 0.11 where station 2 (1st wet) had higher values and station 1(fresh water) had relatively lower value (Table 1). ANOVA result found that Cd were significantly different (*p* < 0.05) among the stations sampled.

The value of chromium was seen in the station2 (1st wet) 0.21 mg/L. and 0.17 from station2 (2nd ) .Statistical Analysis using ANOVA) was found that there was no a mean difference (*P* > 0.05) between Chromium concentration in the stationed sample.

Iron ranges from 0.95 to 1.35 mg/L, station 3(effluent) had 0.95 value and station 2(1st wet) had highest value 1.35 mg/L Fe. Using t-test (2-tailed analysis) it was found that the iron were not significantly different (*p* > 0.05) before and after wetland.

Manganese ranges from 0.0026671 to 0.033, station 1(fresh water) had 0.0026671 value and station 3(effluent) had highest value 0.033. Using t-test (2-tailed analysis) it was found that result showed that the Mn were significantly different before and after wetland.

Lead fluctuated between 0.00206 and 0.002347 mg/L Pb where station 2(2nd wet) had higher values and station 1 and 2(1st ) both had relatively the lower values. The mean toxic metals level of the effluents was out of the EEPA and World Bank permissible limit except Pb [13]. Statistical analysis using ANOVA indicated that there were significant difference (*p* < 0.05) in all the values of among the stations sampled shown except Cr, Mn and Pb.

Most of the results from this study indicated a positive correlation between before and after and between 4 sample stations. This positive correlation can indicate that as the presence of organic matter dissolved solids and suspended solids in water would increase the bacterial load [2]. also reported that the presence of organic matter in the water attribute for the development of microorganisms.

Comparison of GMF effluent concentration of selected parameters obtained in this study against the limit set by WHO regulation [11–13] for safe disposal of malt and beer effluent to surface water is observed as follows: The average value of Fe (0.95), Cd (0.1067), Cr (0.2083) are out of the WHO regulation 0.3, 0.003 and 0.05 respectively. Alkalinity M (570) is out of WHO regulation (400) [13]. All other parameters (ammonia, alkalinity P, conductivity, total hardness, nitrate, nitrite, sulfate. sulfite. phosphate, pH, Mn, Pb, chloride, TDS, and TS) were below the WHO permissible limit [13]. Comparison between Assela and Gondar malt 1st wet step steep waste and treated effluent by different biological method for selected parameters is as shown below (Table 2).

**Table 2:** Comparison of this study with Assela malt with some selected parameters

Parameter	1st wet step steep waste conc.		treated effluent conc.		% Removal efficiency	
	Assela [1, 2]	This study	Assela [1, 2]	This study	Assela [1, 2]	This study
BOD	635.7	732	15.7	465	97.1	36.475
NH_3_-N	12.7	0.5567	8.8	ND	30.7	99.99
NO_3_^−^-N	35.7	3.57	24.5	0.87	31.3	75.63
pH	6.1	7.16557	5.67	7.97	7.05	-11.24
PO_4_^3−^	13.2	404.0557	8.1	15.4	38.6	96.188
SO_4_^2−^	126.25	83	38.25	6	38.25	92.77

As the result of the physicochemical parameters analyzed in this study shows that the effluent discharged from Gondar malt Factory most parameters meet the permissible limit set by EEPA and World Bank for safe discharge to surface water with removal efficiency of above (> 85% NH_3_, Cl_2_free, color, PO_4_^HR^, SO_4_^2−^, TH, NO_3_^−^, NO_2_.^−^), (> 57% Alkalinity P, Turbidity, PO_4_^LR^.), (> 30%BOD, TDS, TSS, TS, Fe, conductivity) (Table 2). This means that Gondar malt Factory uses wastewater treatment system in which the wastewater pass biological (wetland) treatment systems before discharged into ‘Shinta’ River.

Comparison of GMF effluent concentration of selected parameters obtained in this study with that of a Brewery industrial wastewater treatment mesocosm horizontal subsurface flow constructed wetland effluent planted with *C.indica*,* P.karka and C.giganta* is shown in Table 3.

**Table 3:** Comparison of pollutant concentration from effluents by this study and that of constructed wetland planted with different plants for brewery waste treatment

Parameters	This study (treated effluent mean conc.	Brewery industrial wastewater treatment mesocosm HSSFCW effluent [33]		
		*C.indica*	*P.karka*	*C.giganta*
pH	7.97	***6.2***	***6.3***	***6.5***
Temperature	23	***20.9***	***20.7***	***21.8***
Ammonia	ND	22	22	24
BOD	465	453	421	443
Conductivity	749	1218	1330	1279
Nitrate	0.87	19	18	22
Phosphate ^HR^	15.4	13	8	13
TSS	116	40	32	43

The mean effluent pH, temperature, BOD, phosphate HR and TSS of this study were higher than that of a Brewery industrial wastewater treatment mesocosm horizontal subsurface flow constructed wetland effluent planted with *C.indica*,* P.karka and C.giganta.* However, the mean conductivity and nitrate of the studied effluent were lower than that of a Brewery industrial wastewater treatment mesocosm horizontal subsurface flow constructed wetland effluent planted with *C.indica*,* P.karka and C.giganta* [33] which might be due to the differences in the type of wetland leading for the difference in treatment efficiencies.

## Conclusions and recommendations

The results of the physicochemical analysis (pH, Temperature, BOD, TDS, TSS) showed that relatively high mean values were recorded in Station 2 than Station 1 (fresh water) and stations 3(effluent). The relative high mean values of the physicochemical parameters in Station 2 can be attributed to the nature of malt steeping operation. The results also revealed that the parameters NH_3_, Cl_2_free, hardness, color, PO_4_^HR^, SO_4_^2−^, TH, NO_3_^−^, NO_2_ meet the permissible limit set by EEPA and World Bank for discharge into surface water. The result shows that the wetland plays a great role in the removal of pollutants where the best performance was obtained at removal efficiency of 96.188% PO_4_^HR^_,_75.63% Nitrate, > 99% Cl_2,_ ammonia and nitrite 99.99%, 92.77% sulfate,84.36% Total hardness,87.43% color, and for others it is ranged between 30 and 60%. Therefore, the study concluded that GMF wetland was almost effective and had potential in treatment of the wastewater from the discharging facilities (especially for nutrients, alkalinity P, hardness, color and chloride).

This indicates the use of GMF wetland as a wastewater treatment plant is helping in the wastewater treatment effectively for safe discharge of the wastewater into the Shinta River. From this study it is recommended that the use of wetlands is very important for adequate waste disposal facilities for other similar factories. In this study only physicochemical parameters were studied hence, it is also recommended that further studies to be conducted on the microbiological quality of the waste water before discharged in to Shinta River.

## Data Availability

All data generated and analyzed are included within this research article.
